# Supplementary material for: A potent SARS-CoV-2 neutralising nanobody shows therapeutic efficacy in the Syrian golden hamster model of COVID-19
Source: Nat Commun. 2021 Sep 22;12:5469. doi: 10.1038/s41467-021-25480-z (PMC8458290; doi:10.1038/s41467-021-25480-z)
Supplement: Supplementary file 1 — Supplementary information [file 41467_2021_25480_MOESM1_ESM.pdf]

A potent SARS-CoV-2 neutralising nanobody shows therapeutic efficacy in the Syrian golden hamster model of COVID-19

Jiandong Huo Halina Mikolajek, Audrey Le Bas, Jordan J. Clark, Parul Sharma, Anja Kipar, Joshua Dormon, Chelsea Norman, Miriam Weckener, Daniel K. Clare, Peter J. Harrison, Julia A. Tree, Karen R. Buttigieg, Francisco J. Salguero, Robert Watson, Daniel Knott, Oliver Carnell, Didier Ngabo, Michael J. Elmore, Susan Fotheringham, Adam Harding, Lucile Moynié, Philip N. Ward, Maud Dumoux, Tessa Prince, Yper Hall, Julian A. Hiscox, Andrew Owen, William James, Miles W. Carroll, James P. Stewart, *James H. Naismith and *Raymond J. Owens

Supplementary information

Supplementary Fig. 1 Nanobody sequences

Supplementary Fig. 2 Analysis of nanobody binding by Isothermal calorimetry

Supplementary Fig. 3 Nanobody-RBD interfaces

Supplementary Fig. 4 Cryo-EM of the Spike-nanobody C5 complex

Supplementary Fig. 5 Cryo-EM of two different Spike-nanobody complexes

Supplementary Fig. 6 Nanobody binding kinetics

Supplementary Fig. 7 Plaque Reduction Neutralisation assays

Supplementary Fig. 8 Histopathological semiquantitative analysis and presence of virus RNA (RNAScope ISH S-gene) in the nasal cavity of control and nanobody treated animals.

Supplementary Fig. 9 Histopathology and image analysis of lung in control and nanobody treated animals.

Supplementary Fig. 10 Macrophage driven inflammatory response after C5 trimer neutralisation of SARS-CoV-2 in Syrian hamster model.

Supplementary Fig. 11 Viral antigen sequestration in macrophages in the focal infiltrates after C5 trimer neutralisation of SARS-CoV-2 in Syrian hamster model.

Supplementary Table 1: PCR primer sequences

Supplementary Table 2: DNA and amino acid sequences of C1, C5, F2 and H3 nanobodies

a

Clone ID	CDR1-IMGT	CDR2-IMGT	CDR3-IMGT	Inhibition%
F2	GRTFHSYV	ISWSSTPT	AADRGESYYYTRPTEYEF	76
H7	GRTFHSYV	ISWSSTLT	AADRGESYYYTRPTEYEF	74
C1	GFTNDFYS	LSVSDNTP	AAGRFAGRDTWPSSYDY	62
C5	GVTLGRHA	IRTFDGIT	ALGVTAACSDNPYF	62
H3	GRTFSTYS	MRWTGSST	AITTIVRAYYTEYTEADFGS	56
H5	GRTFSMAR	INWSSGSI	AVQFGIRGYLDGYDY	47
H2	GRTLSMYP	ISWSGDNT	AARTRAHMYSTTYPYASEYDY	22
F8	GVTLGRHA	IRTFDGIT	ALRVTGACSDNPYF	21
G4	GRTFSSIV	IDWSGGGT	AADRGHSYYYTRPTEYDY	20
B5	GGTFRPYT	ISRLTGTT	AATTNTAVASTTSVYGY	18
D1	ERTSISSV	IRPGIGNT	AIAMSLNNLGAGLDTRVYDY	16
H8	GRTITAYT	ISKSSDST	AAGSWYGRDSDPAGYDY	15
G6	GRALNSLI	IVWSDEFT	AGRYGNLITQNQNEYQY	12
A5	GRTFSRHT	IRWLGGG	YCAAAPAGFVGPTMERYPYEY	5
E1	GRTFDTTR	IFRNTGTT	AAGRFSAAPLTRSTAFES	-20
H4	GSTLDNYS	FSRRYGAP	ATRSGPYCTTSVSDFDD	-21

b

```
   1
          FR1                      CDR1         FR2            CDR2              FR3
C1 QVQLVESGGGLVQPGGSLRLSCAASGFTNDFYSIAWFRQAPGKEREGVSWLSVSDNTPTYVDSVKDRFTISRHNANNTVYLQMNMLKPEDT
C5 QVQLVESGGGSVQAGGSLTLSCVASGVTLGRHAIGWFRQAPGKERERVSCIRTFDGITSYVESTKGRFTISSNNAMNTVYLQMNSLKPEDT
F2 QVQLVESGGGLVQAGGSLRLACIASGRTFHSYVMAWFRQAPGKEREFVAAISWSSTPTYYGESVKGRFTISRDNAKNTVYLQMNRLKPEDT
H3 QVQLVESGGGLVKTGGSLRLSCAASGRTFSTYSMGWFRQAPGKEREFVAGMRWTGSSTFYSDSVKGRFTVSRNNAKDTVYLHMNSLKPEDT
   ********** *:.**** *:* *** *    : :.*********** *: :    .   . * :*.*.***:* .** :****:** ******

   FR3          CDR3          FR4
C1 AIYYCAAGRFAGR---DTWPSSYDYWGQGTQVTVSS
C5 AVYFCALGVTAAC------SDNPYFWGQGTQVTVSS
F2 AVYFCAADRGESYYYTRPT--EYEFWGQGTQVTVSS
H3 AVYYCAITTIVRAYYTEYTEADFGSWGQGTQVTVSS
   *:*:**                  .  ***********

  92
```

Supplementary Fig. 1 Nanobody sequences

(a) The CDR sequences of the 15 unique CDR sequences identified from 93 picked VHH clones ranked according to % inhibition of binding to immobilised RBD by soluble RBD. The selected clones are highlighted (b) amino acid alignment of alignment of selected nanobodies (C1, C5, F2 and H3).

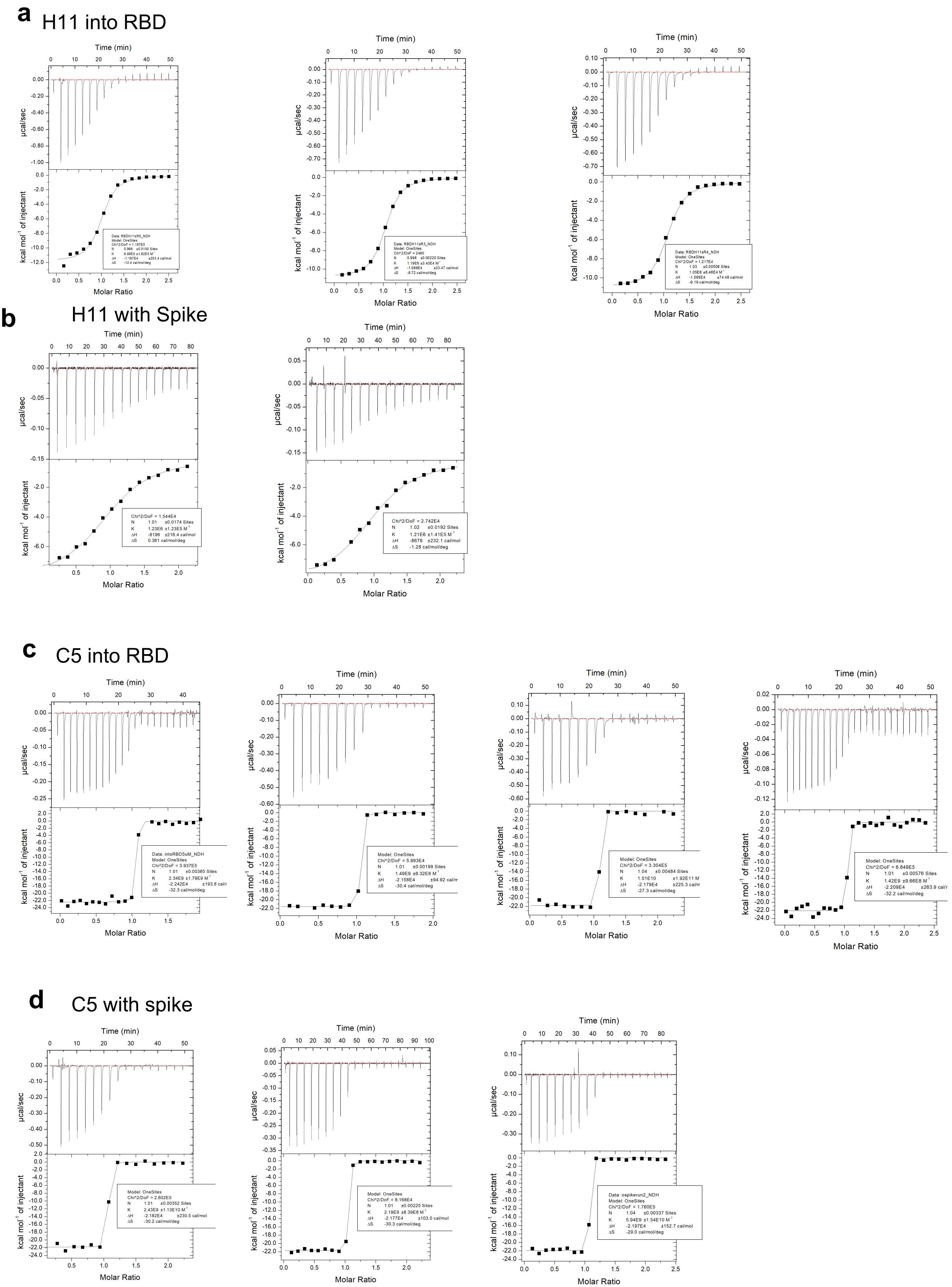

Supplementary Fig. 2

e

200 µM C5 with 20 µM RBD + 100 µM H11

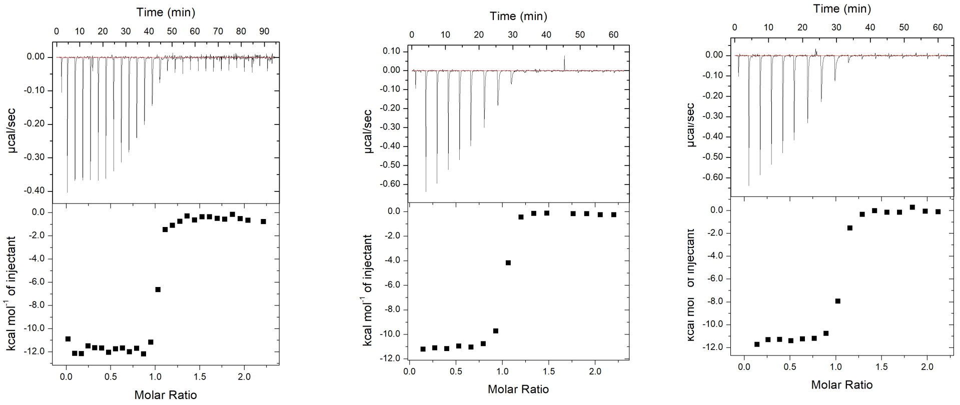

f

61 µM C5 into 6 µM Spike + 186 µM H11

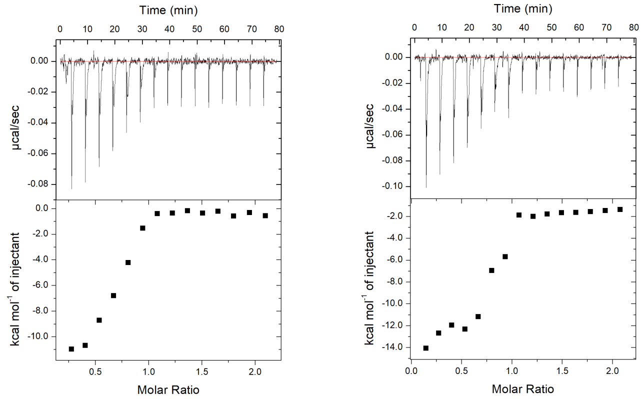

g

200 µM C5 into 20 µM RBD + 100 µM H11 | C5 into RBD | H11 into RBD

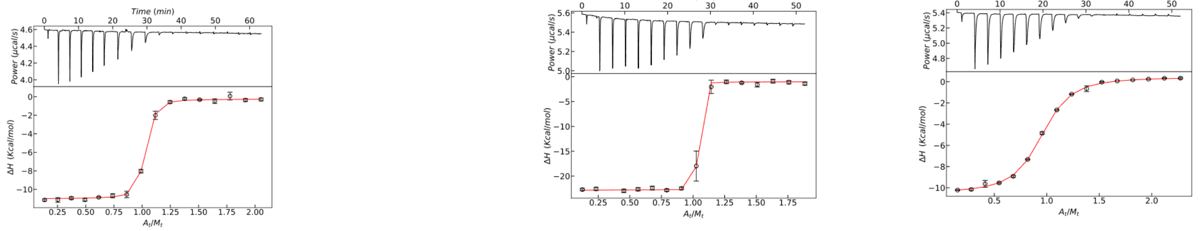

Kd (C5) =213 pM; Kd (H11)=854 nM

K_{aC5} = 4.68e^{+9}± 1.6e^{+8}, ΔH_{C5}=-21.6 e^{+3} ± 1.17e^{+1}

K_{aH11} = 1.12e+^{6} ±9.6e^{+3}, ΔH_{11}=-11.04e^{+3} ±6.95e^{+1}

h

61 µM C5 into 6 µM Spike + 186 µM H11 | C5 into spike | H11 into Spike

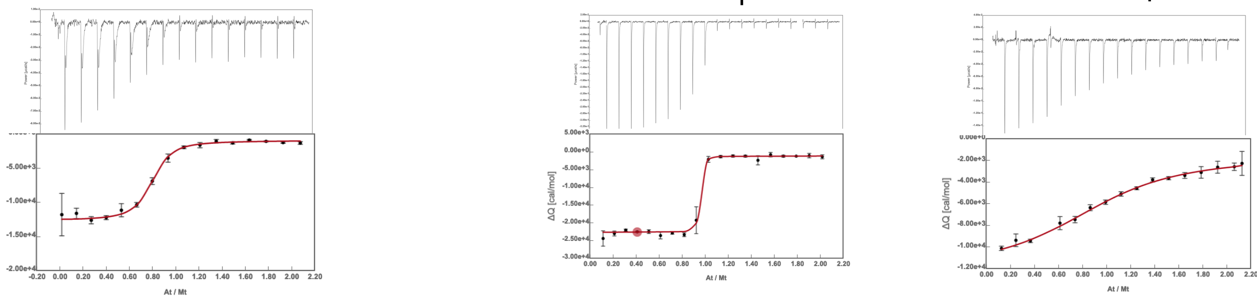

Kd (C5) =345 pM, Kd (H11)=1170 nM

K_{aC5} = 2.897e^{+9}± 4.57e^{+7}, ΔH_{C5}=-21.2 e^{+3} ± 2.3e^{+1}

K_{aH11} = 8.485e+^{5} ±1.33e^{+4}, ΔH_{11}=-9.8e^{+3} ±4.2e^{+2}

Supplementary Fig. 2

i

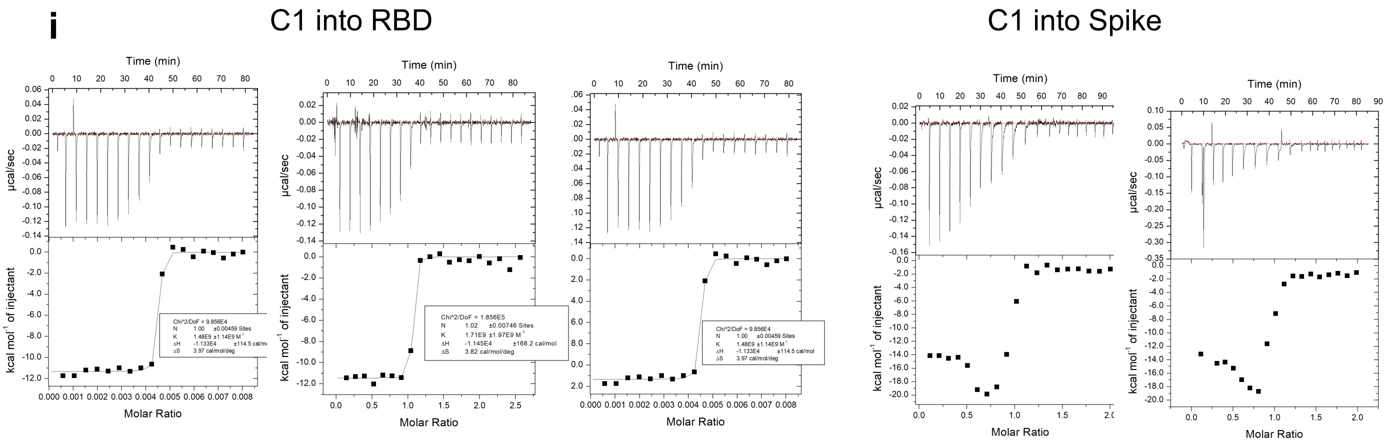

j

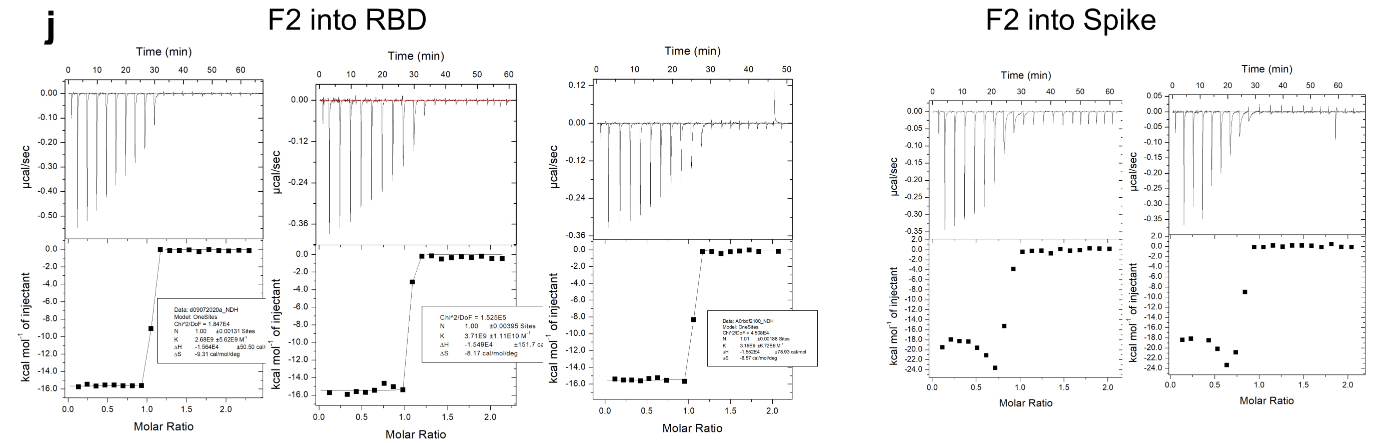

Supplementary Fig. 2 Analysis of nanobody binding by Isothermal calorimetry

(a) Titration of the H11 (472, 465, 460 µM) nanobody into RBD (47, 42, 38 µM) (three independent measurements), **(b)** Titration of the H11 nanobody (67 µM) into spike protein (6.8 µM) (two independent measurements), **(c)** Titration of the C5 nanobody (50, 89, 110 and 25 µM) into RBD (4.6, 9.7, 10 and 2.2 µM) (four independent measurements), the very tight binding make fitting thermodynamic parameters prone to large error, **(d)** Titration of the C5 nanobody (83, 67, 67 µM) into spike protein (7.6, 6.1, 5.8 µM) (three independent measurements), the very tight binding make fitting thermodynamic parameters prone to large error **(e)** Nanobody C5 (200, 194, 193 µM) was titrated into RBD (18, 19, 18 µM) where H11 (100 µM) was already present (three repeats). No binding curve was fitted, **(f)** Nanobody C5 (61, 68 µM) was titrated into spike protein (6, 6 µM) where H11 (180, 104 µM) was already present (two repeats). No binding curve was fitted. **(g)** AFFINImeter[60] simultaneous fitting (red line) of thermodynamic parameters for C5 into RBD in presence of H11, C5 into RBD and H11 into RBD from the three independent measurements. **(h)** AFFINImeter simultaneous fitting (red line) of thermodynamic parameters for C5 into spike protein in presence of H11, C5 into Spike and H11 into spike protein. **(i)** Three independent experiments of C1 (105, 49, 49 µM) titrated into RBD (9.9, 4.7, 3.9 µM respectively). The very tight binding means the calculation of K_D has large errors. Two experiments of C1 (64, 63µM) titrated into spike protein (6.4, 6.4 µM respectively). The titration shows that complex conformational changes occur, and it was not possible to reliably fit a binding constant. **(j)** Three independent experiments of F2 (150, 100, 80 µM) titrated into RBD (13, 10, 7.8) µM respectively). The very tight binding means the calculation of K_D has large errors. Two experiments of F2 (86 µM) titrated into spike protein (8.6µM respectively). The titration shows that complex conformational changes occur, and it was not possible to reliably fit a binding constant. The AFFINImeter software automatically calculates statistical errors as the standard deviation of the parameters obtained by repeat fitting as many times as experimental points are available in the target data series.

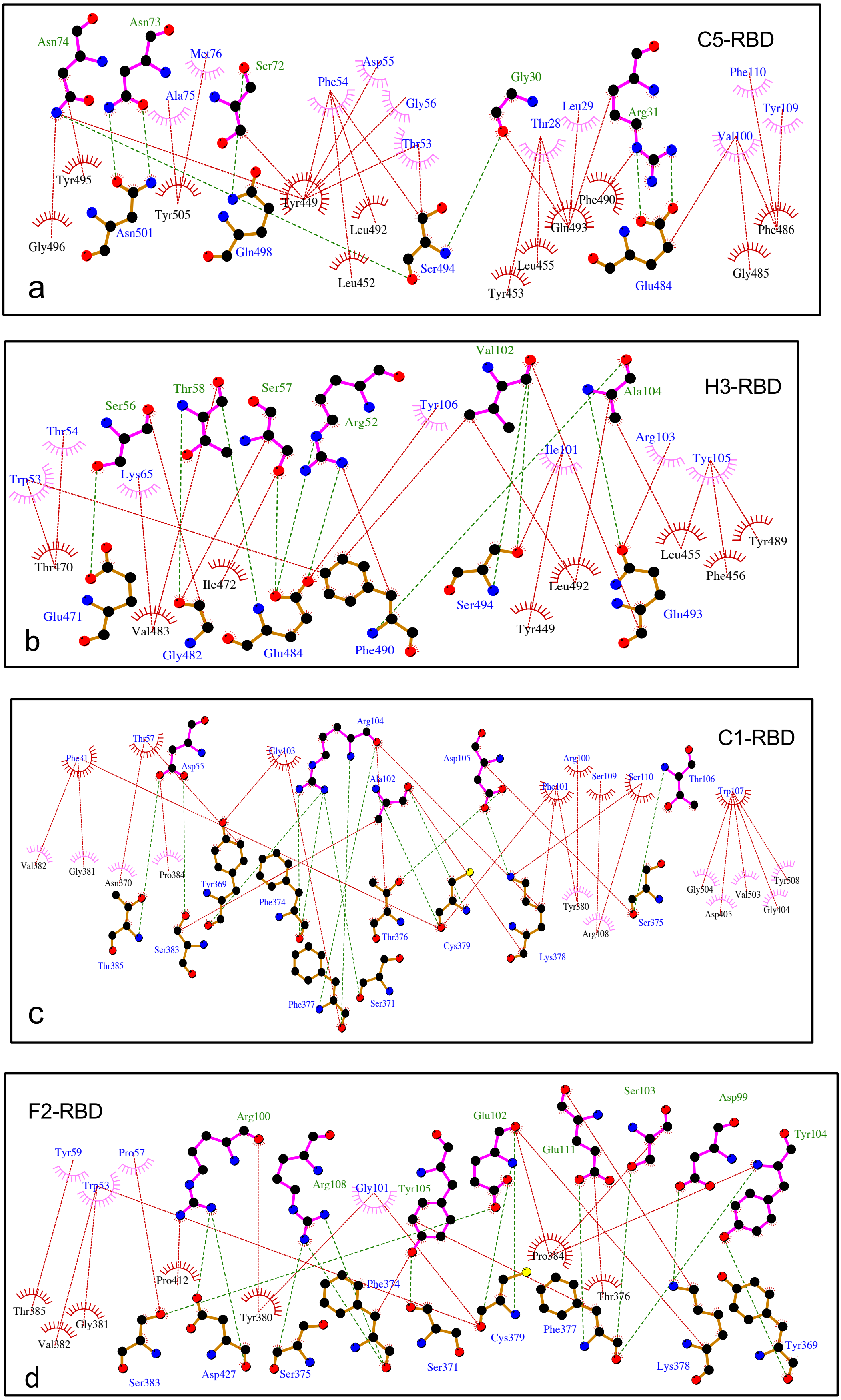

Supplementary Fig. 3 Nanobody-RBD interfaces

LigPlots[1] of the interaction between C5, H3, F2 and C1 (residues shown in purple, top) and RBD (residues shown in brown, below). Hydrogen bonds are shown as green dashes and van der Waals interactions as red dashes.

[1]Wallace A C, Laskowski R A, Thornton J M (1996). LIGPLOT: a program to generate schematic diagrams of protein-ligand interactions. Protein Eng., 8, 127-134.

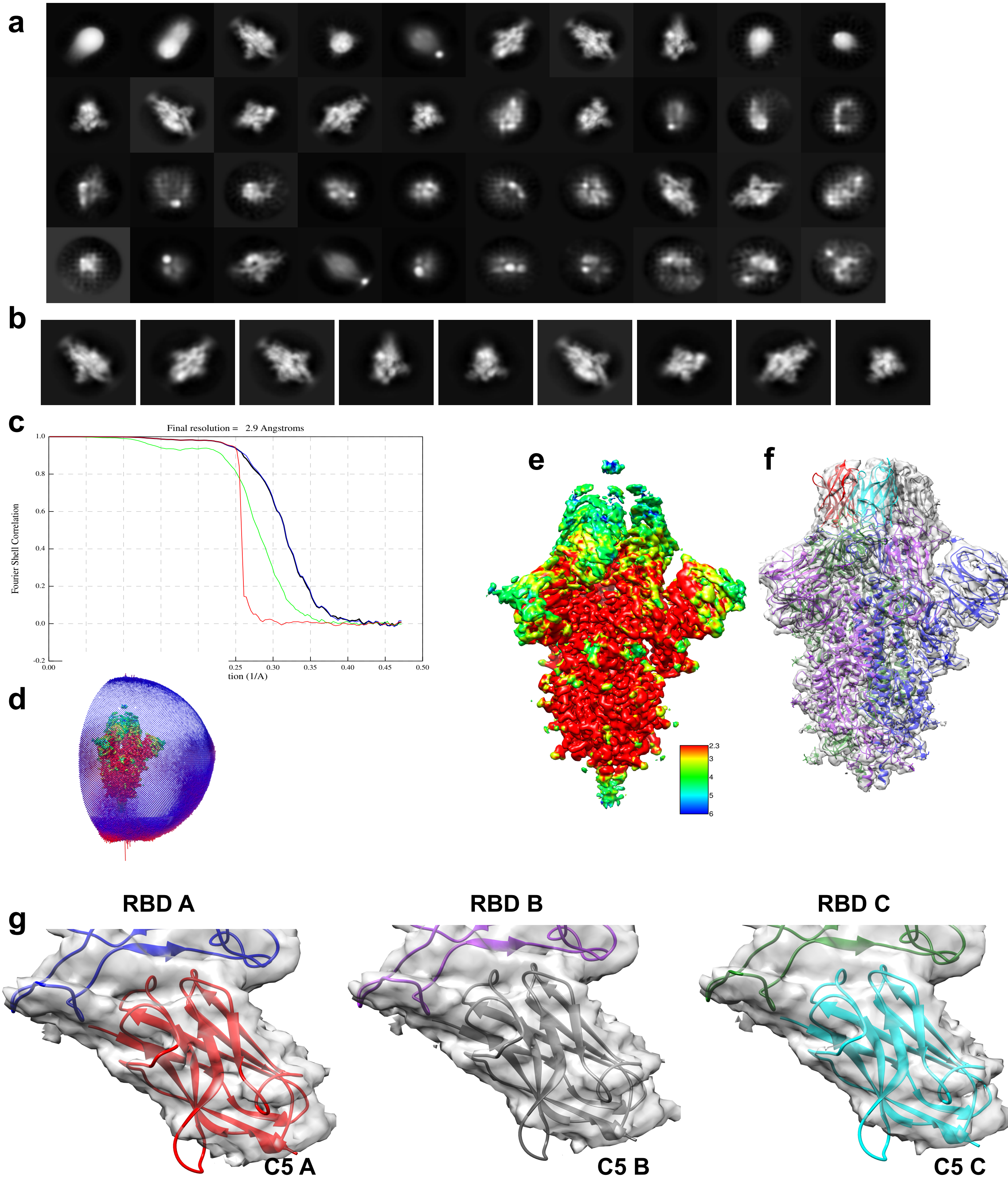

Supplementary Fig. 4 Cryo-EM of the Spike-nanobody C5 complex

(a) Unbiased 2D class averages of the Sars-CoV-2 spike-nanobody C5 complex, (b) 2D class averages selected for further processing. (c) Estimated map resolution using FSC criteria, (d) Particle orientation distribution for the final map. (e) Final cryo-EM map (coloured according to local resolution). (f) Ribbon diagram of the complex (cryo-EM map shown in grey and contoured at 5 **σ** in chimera). (g) Cryo-EM density with ribbon for each of the three C5 nanobodies bound to RBD, contoured at 5 **σ** in chimera. The amplitudes in the cryo-EM map used in panels f-g were scaled based on the refined coordinates using LocScale64 .

a

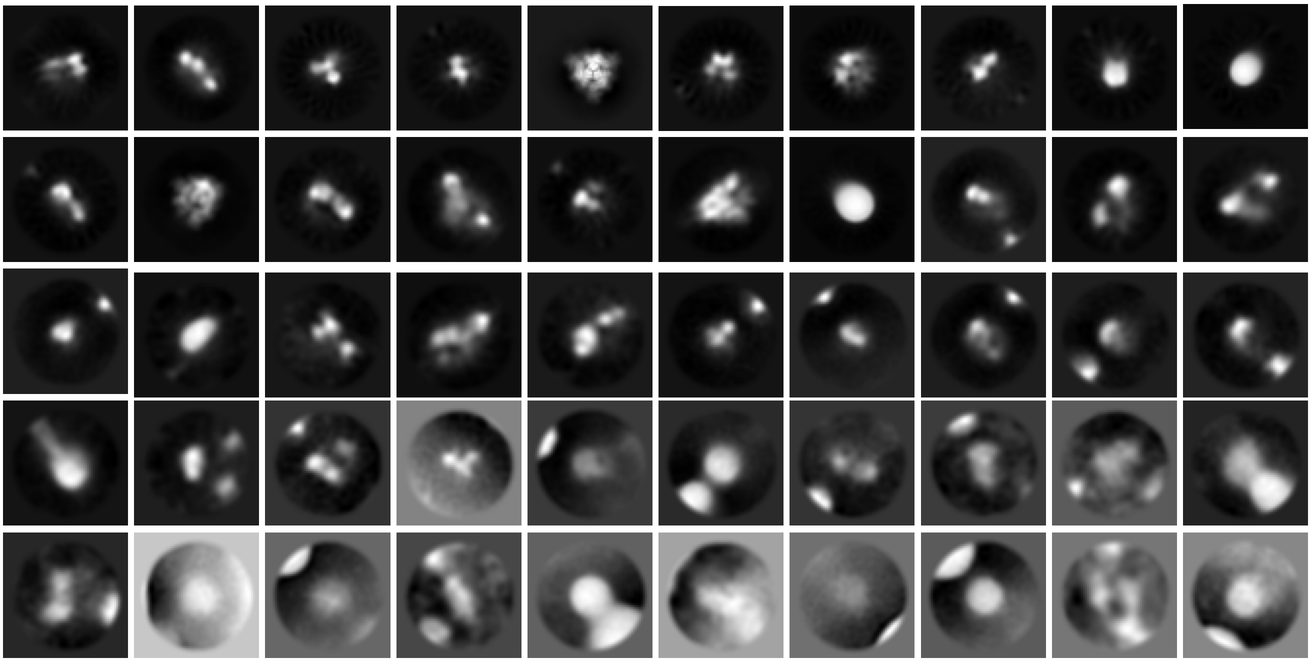

b

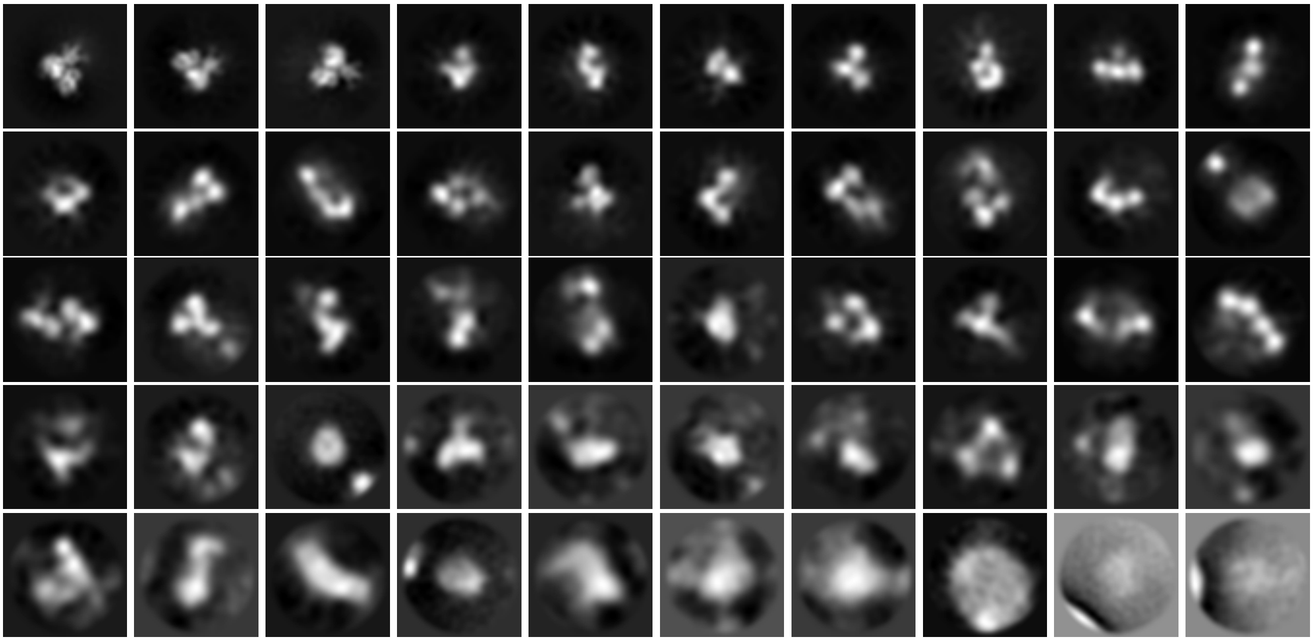

Supplementary Fig. 5 Cryo-EM of two different Spike-nanobody complexes
(a) Unbiased 2D class averages of the Sars-CoV-2 spike-nanobody C1 complex. These show heterogenous particles without any indication of trimeric spike arrangement. **(b)** Unbiased 2D class averages of the Sars-CoV-2 spike-nanobody F2 complex. These show heterogenous particles without any indication of trimeric spike arrangement.

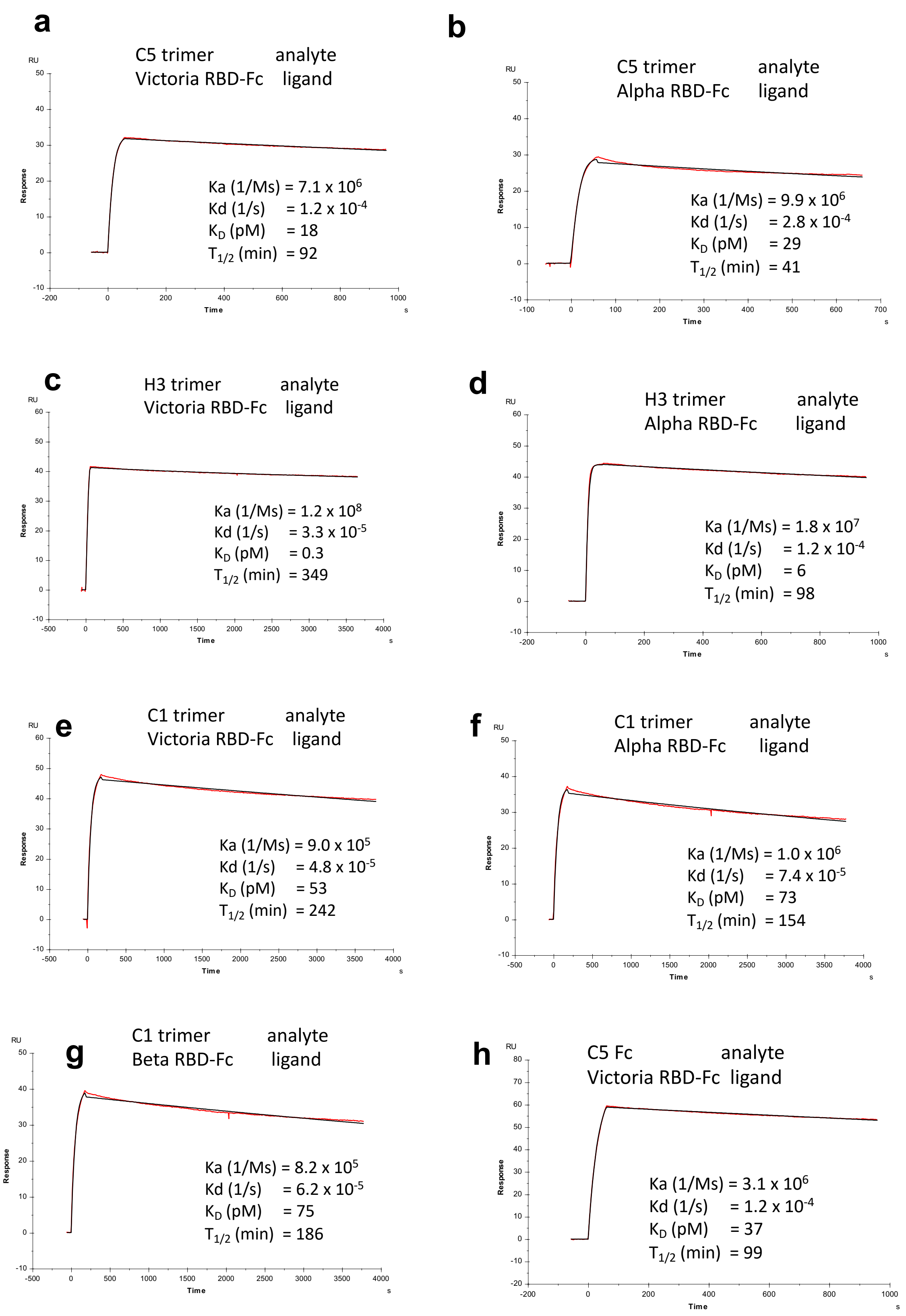

Supplementary Fig. 6 Nanobody binding kinetics

SPR Sensorgrams showing binding kinetics of trimeric C5 to (a) Victoria and (b) Alpha RBDs, trimeric H3 binding to (c) Victoria, (d) Alpha RBDs and trimeric C1 binding to (e) Victoria, (f) Alpha and (g) Beta RBDs. (h) C5-Fc binding to Victoria. A single injection of C1 / C5 / H3 trimer was performed over the huIgG1 Fc fusion of RBDs (a-g), and a single injection of C5-Fc over the biotinylated Victoria RBD (h), to evaluate the kinetics of interactions.

a

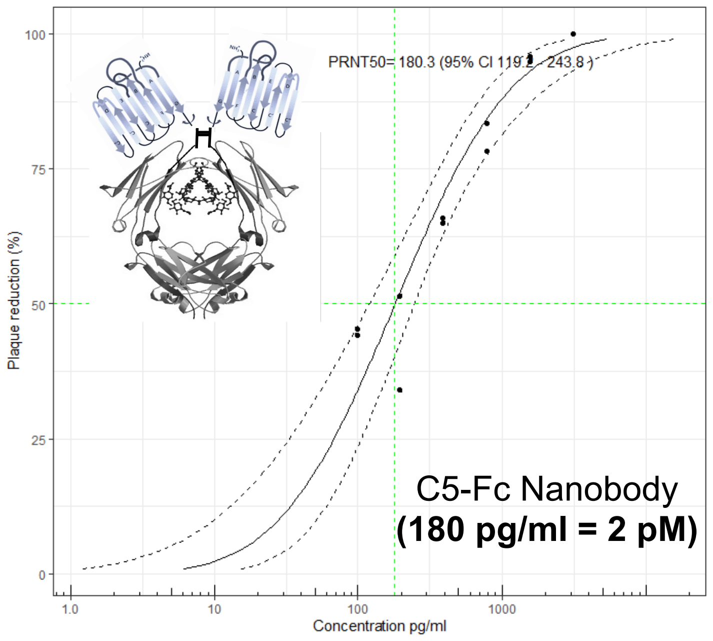

b

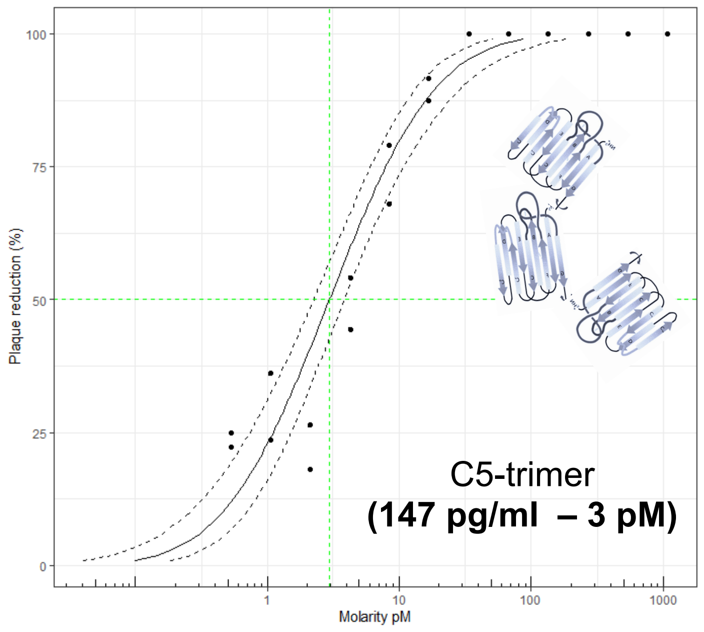

Supplementary Fig. 7 Plaque Reduction Neutralisation assays

The percentage reduction in plaques arising from virus is plotted against increasing (left to right) concentration of (a) C5-Fc (b) C5 trimer, shown schematically as insets. The confidence intervals are shown as dashed lines.

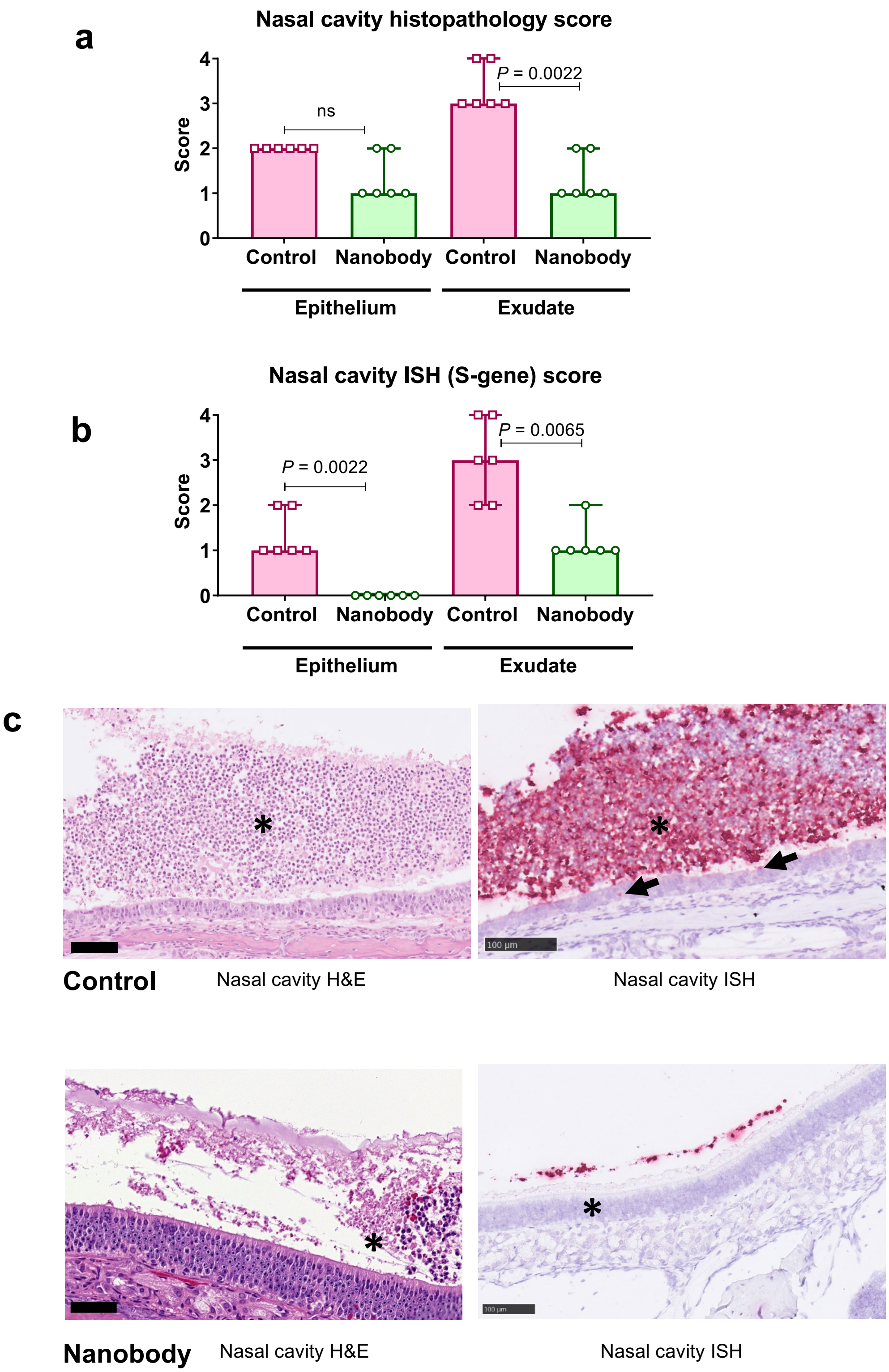

Supplementary Fig. 8 Histopathological semiquantitative analysis and presence of virus RNA (RNAScope ISH S-gene) in the nasal cavity of control and nanobody treated animals. (a) A significant reduction in the presence of inflammatory exudates and the presence of virus RNA (*) was observed in the treatment group. (b) Minimal to mild necrosis of the epithelium was observed in both groups. Data are from n = 6 biologically independent animals, box and error bars show median and 95% C.I. Mann-Whitney's U test for median comparisons two-sided using Prism GraphPad. (c) representative images of nasal cavity stained with H&E and ISH (RNAScope for virus RNA). The presence of virus RNA in epithelial cells was only observed in control animals (arrows). Bar = 100 µm. Large sections covering the whole nasal cavity of the animal reading/interpretation in each pathology study at PHE always include double reading (for intra or interpersonal variation) and peer review of some slides by a different pathologist.

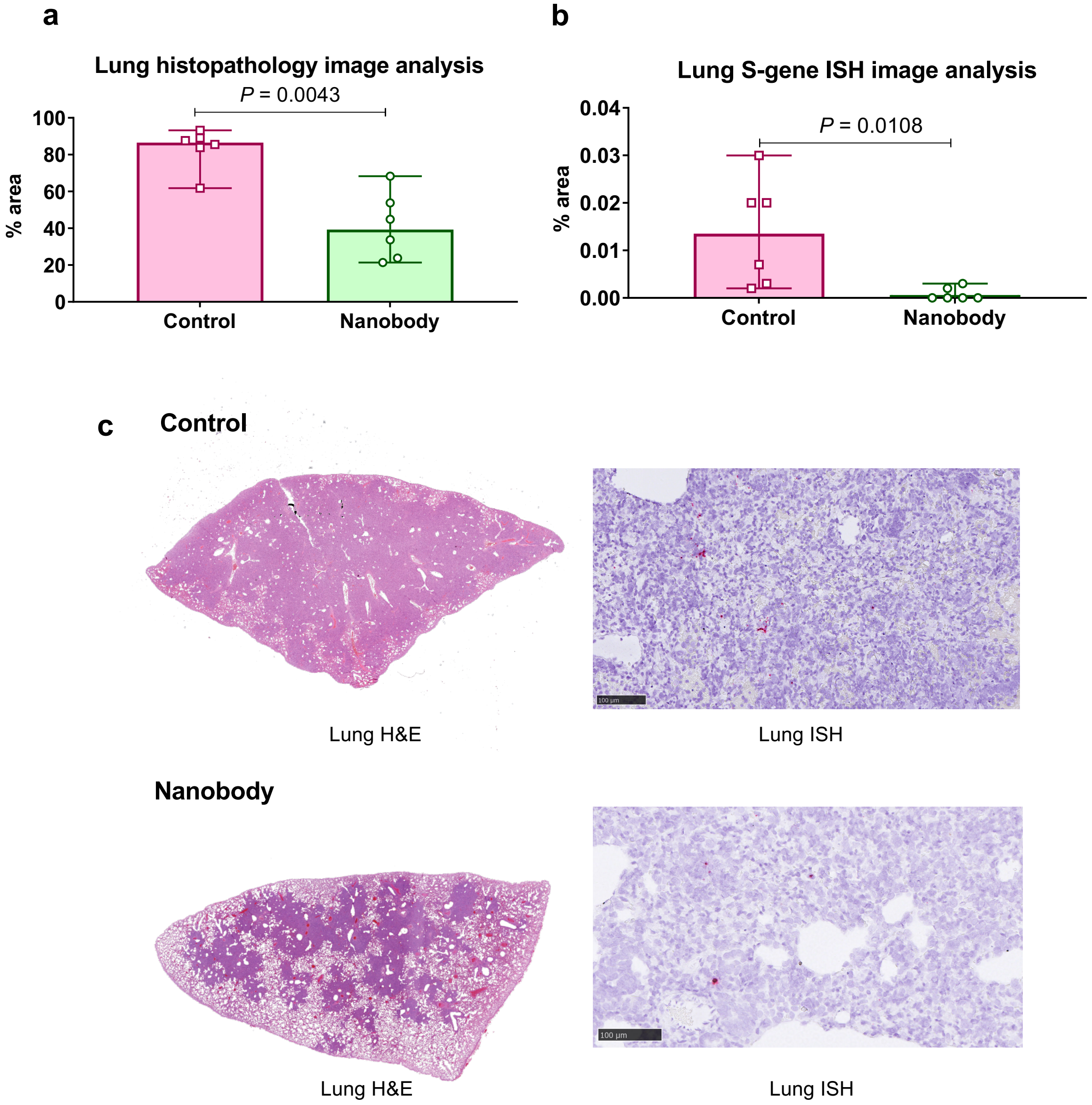

Supplementary Fig. 9 Histopathology and image analysis of lung in control and nanobody treated animals.

(a) lung histopathology image analysis showing the percentage of the lung area affected and (b) percentage of lung area with viral RNA-ISH staining with representative images of lung stained with HE showing larger areas of lung consolidation in the non-treated control group, and with RNAScope ISH (S-gene) showing larger areas of positive stained lung tissue in the control group. Data are from n = 6 biologically independent animals, box and error bars show median and 95% C.I. Mann-Whitney's U test for median comparisons two-sided using Prism GraphPad (c) representative images of lung stained with H&E and ISH (RNAScope for virus RNA) Bar = 100 μm. We have not made section/staining repeats. Images are of a single large section of the lung left lobe. Reading/interpretation in each pathology study at PHE always include double reading (for intra or interpersonal variation) and peer review of some slides by a different pathologist.

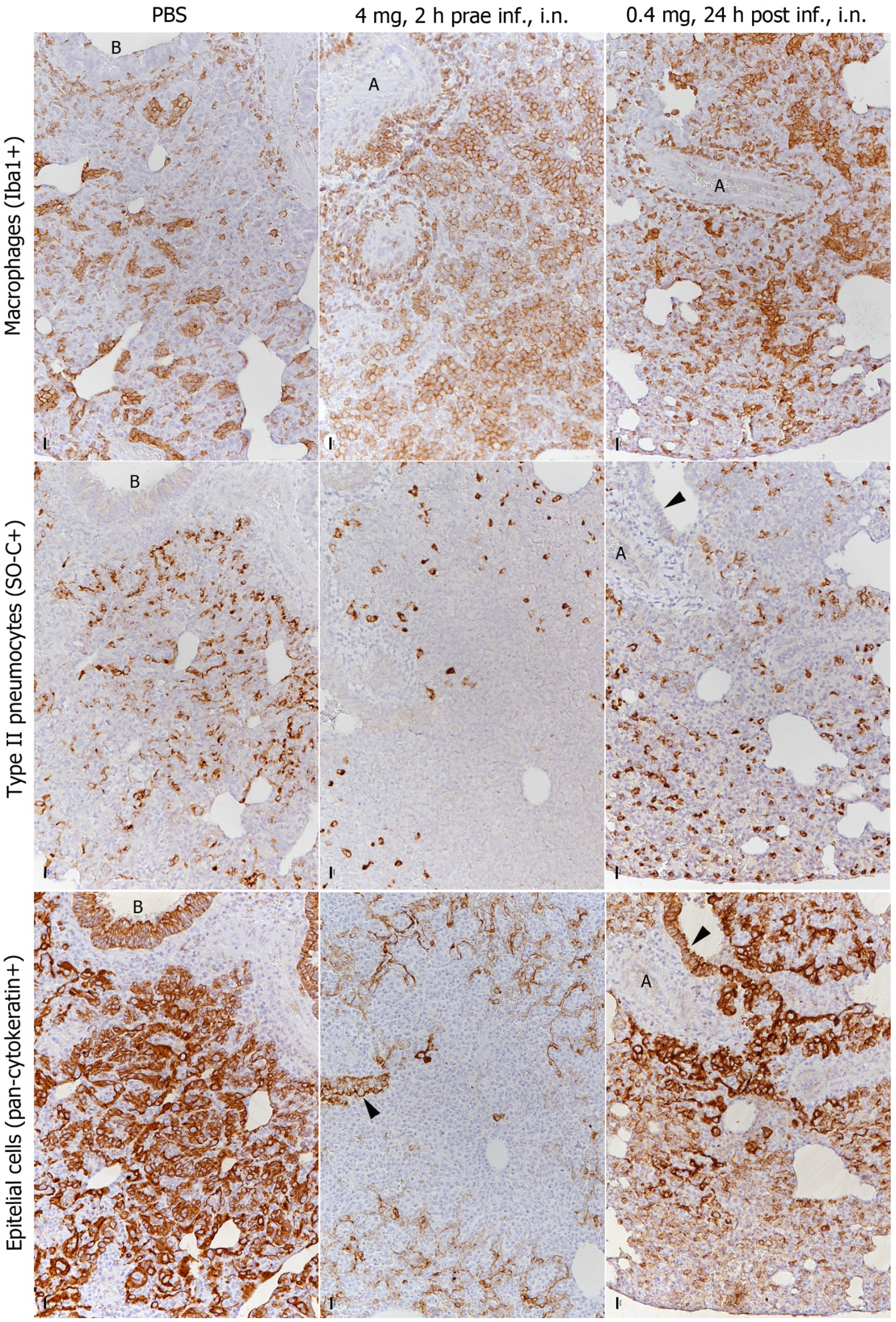

Supplementary Fig. 10 Macrophage driven inflammatory response after C5 trimer neutralisation of SARS-CoV-2 in Syrian hamster model.
Lungs of hamsters infected intranasally with 10^4 PFU/100 μl SARS-CoV-2 and euthanized at day 7 post infection. Animals had been untreated prior to infection (PBS) or treated with 4 mg/kg C5 intranasally at 2 h prae infection (h prae inf) or 0.4 mg/kg C5 intranasally at 24 h post inf (h post inf). Staining for macrophages (Iba1+) showed clusters of macrophages in the consolidated areas in the untreated (PBS) animals and a very intense macrophage infiltration in the treated animals. In untreated animals there are numerous activated and hyperplastic type II pneumocytes (SP-C+); these are less numerous in hamsters treated with the low C5 trimer dose and replaced by the macrophage dominated infiltrate in animals treated with the high dose at 2 h prae inf. Staining for cytokeratins shows that in untreated animals the consolidated areas are dominated by hyperplastic bronchiolar epithelium. The hyperplasia is less intense in hamsters treated with the low C5-trimer dose and limited to the periphery of the infiltrates in animals treated with the high dose at 2 h prae inf. B – bronchiole (arrowhead: normal bronchiolar epithelium); A – artery. Immunohistology, hematoxylin counterstain. Bars = 20 µm. Images are representative of n = 6 independent biological samples.

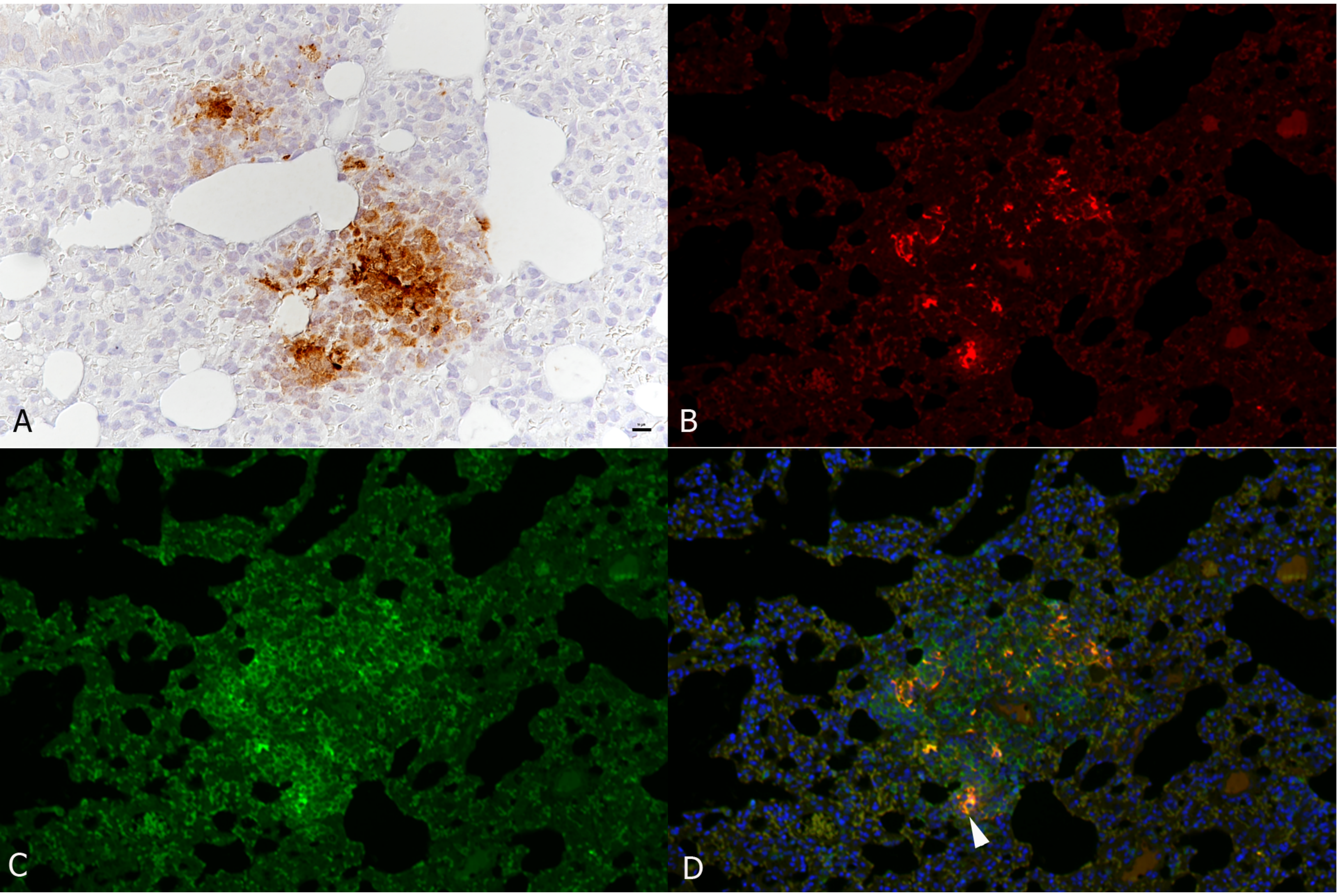

Supplementary Fig. 11 Viral antigen sequestration in macrophages in the focal infiltrates after C5 trimer neutralisation of SARS-CoV-2 in Syrian hamster model. Lung of hamster treated with 4mg/kg C5 intranasally at 2 h prae infection, infected intranasally with 10^4 PFU/100 μl SARS-CoV-2 and euthanized at day 7 post infection. **A**. Small focal consolidated area with SARS-CoV-2 N expression in intact and degenerate cells. Immunohistology, hematoxylin counterstain. **B**. SARS-CoV-2 N expression (red fluorescence) in consecutive section. **C**. The consolidated area contains abundant macrophages (Iba1+; green fluorescence) in consecutive section. **D**. Combined image, confirming viral antigen expression in macrophages (arrowhead) in the lesion. Panel A scale Bar = 30 µm. All images at same magnification. Images are representative of n = 6 independent biological samples.

Primer ID	Sequence
CALL_GSP	5’-CCTGCGGCTCCCGGGTCTGCCCTTTGGCC-3’
CALL_001	5’-GTCCTGGCTGCTCTTCTACAAGG-3’
CALL_002	5’-GGTACGTGCTGTTGAACTGTTCC -3’
VHH_For	5’-GTTATTACTCGCGGCCCAGCCGGCCATGGCCGATGTGCAGCTGCAGGAGTCTGGRGGAGG-3’
VHH_Rev_IgG2	5’-GGTGATGGTGTTGGCCTCCCGGGCCGGCCGCTGGTTGTGGTTTTGGTGTCTT-3’
VHH_Rev_IgG3	5’-GGTGATGGTGTTGGCCTCCCGGGCCGGCCGCGGAGCTGGGGTCTTCGCTGTG-3’
OmA_exp_F	5’-CTACCGTAGCGCAAGCTCAGGTGCAGCTGGTCGAGTCTGGGGGA-3’
OmA_exp_R	5’-GGTGATGGTGATGTTTTGAGGAGACGGTGACCTGGGTCCCCTGGCC-3’
TriNb_Neo_F1	5’-GCGTAGCTGAAACCGGCCAGGTGCAGCTGGTGGAGTCTGGG-3’
TriNb_R1	5’-GACTCCACCAGCTGCACCTGGGAGCCAGAACCGCTCCCTGAGGAGACGGTGACCTGG-3’
TriNb_F2	5’-CCCAGGTCACCGTCTCCTCAGGGAGCGGTTCTGGCTCCCAGGTGCAGCTGGTGGAG-3’
TriNb_R2	5’-GACTCCACCAGCTGCACCTGCGACCCGCTACCTGAGCCTGAGGAGACGGTGACCTGG-3’
TriNb_F3	5’-CCCAGGTCACCGTCTCCTCAGGCTCAGGTAGCGGGTCGCAGGTGCAGCTGGTGGAG-3’
TriNb_Neo_R1	5’-GTGATGGTGATGTTTTGAGGAGACGGTGACCTGGGTCCC-3’
TriNb_Neo_F2	5’-GCGTAGCTGAAACCGGCCAG-3’
TTGneo_RBD_F	gcgtagctgaaaccggcccgaatatcacaaatctttgt
TTGneo_RBD_R	GTGATGGTGATGTTTATTTGTACTTTTTTTCGGTCCGCACAC
K417V_F	CAGATCGCGCCGGGCCAAACGGGCGTGATAGCTGACTATAATTATAAG
K417V_R	CTTATAATTATAGTCAGCTATCACGCCCGTTTGGCCCGGCGCGATCTG
E484K_F	ggttcaaccccctgcaatggcgtcAAGGGTTTTAACTGTTACTTCCCAC
E484K_R	GTGGGAAGTAACAGTTAAAACCCTTgacgccattgcagggggttgaacc
N501Y_F	Cagtcatacggatttcaaccaacttacggggttggctatcagccgtaccgc
N501Y_R	gcggtacggctgatagccaaccccgtaagttggttgaaatccgtatgactG
PelB_F	5’-GTTATTACTCGCGGCCCAGCCGGCCATGGCCCAGGTGCAGCTGGTCGAGTCTGGG-3’
PelB_R	5’-GGTCACCGTCTCCTCACACCATCACCACCATCATTAATAAAGGCCAACACCATCACC-3’
AbVec-Fc_F	5’-CTAGTAGCAACTGCAACCGGTGTTCACTCTCAGGTGCAGCTGGTGGAGTCTGGG-3’
AbVec-Fc_R	5’-GATTTGGGCTCGGTCGACGCTGAGGAGACGGTGACCTGGGTCCC-3’
RBD_Fc_R	5’-CAGAACTTCCAGTTTATTTGTACTTTTTTTCGGTCCGC-3’
TriNb_Neo_R2	5’-GTGATGGTGATGTTTTGAGG-3’
RBD_TTGneo_F	GCGTAGCTGAAACCGGCCCGAATATCACAAATCTTTGTCC
RBD_TTGneo-BAP_R	GTCATTCAGCAAGCTATTTGTACTTTTTTTCGGTCCGCACAC

Supplementary Table 1: PCR primer sequences

Nanobody	DNA sequence	Amino acid sequence
C1	CAGGTGCAGCTGGTGGAGTCTGGGGGAG<br>GCTTGGTGCAGCCTGGGGGCTCTCTGAG<br>ACTCTCCTGTGCAGCCTCTGGATTCACT<br>AATGATTTTTATAGCATCGCGTGGTTCC<br>GCCAGGCCCCAGGAAAGGAGCGTGAGGG<br>GGTCTCATGGCTTAGTGTCAGTGATAAT<br>ACCCCAACCTACGTAGACTCCGTGAAGG<br>ACCGGTTCACCATCTCCAGACACAACGC<br>CAACAACACCGTGTACCTGCAAATGAAC<br>ATGCTGAAACCTGAGGACACGGCCATTT<br>ACTATTGTGCAGCAGGACGCTTCGCGGG<br>AAGGGATACTTGGCCCTCGTCCTATGAT<br>TACTGGGGCCAGGGGACCCAGGTCACCG<br>TCTCCTCA	QVQLVESGGGLVQPGGSLRLSCAA<br>SGFTNDFYSIAWFRQAPGKEREGV<br>SWLSVSDNTPTYVDSVKDRFTISR<br>HNANNTVYLQMNMLKPEDTAIYYC<br>AAGRFAGRDTWPSSYDYWGQGTQV<br>TVSS
C5	CAGGTGCAGCTGGTGGAGTCTGGGGGAG<br>GCTCGGTGCAGGCTGGGGGGTCTCTGAC<br>ACTCTCCTGTGTCGCCTCTGGAGTCACT<br>TTGGGACGTCATGCCATAGGCTGGTTCC<br>GCCAGGCCCCCGGGAAGGAGCGTGAGAG<br>AGTCTCGTGTATTAGAACATTTGATGGC<br>ATCACAAGTTATGTAGAGTCCACGAAGG<br>GCCGATTCACCATCTCCAGTAACAATGC<br>CATGAACACGGTGTATCTGCAAATGAAT<br>AGCCTAAACCTGAAGACACGGCCGTTTA<br>TTTCTGTGCACTGGGAGTGACTGCAGCC<br>TGTTCAGATAATCCCTACTTCTGGGGCC<br>AGGGGACCCAGGTCACCGTCTCCTCA	QVQLVESGGGSVQAGGSLTLSCVA<br>SGVTLGRHAIGWFRQAPGKERERV<br>SCIRTFDGITSYVESTKGRFTISS<br>NNAMNTVYLQMNSLKPEDTAVYFC<br>ALGVTAACSDNPYFWGQGTQVTVS<br>S
F2	CAGGTGCAGCTGGTGGAGTCTGGGGGAG<br>GATTGGTGCAGGCTGGGGGCTCTCTAAG<br>CTCGCTTGTATAGCCTCTGGACGCACCT<br>TCCATAGCTATGTCATGGCCTGGTTCCG<br>CCAGGCTCCAGGGAAGGAGCGTGAGTTT<br>GTAGCAGCTATTAGTTGGAGTAGTACAC<br>CGACATACTATGGAGAATCCGTGAAGGG<br>CCGATTCACCATCTCCAGAGACAACGCC<br>AAGAACACGGTGTATCTGCAAATGAACC<br>GCCTGAAACCTGAGGACACGGCCGTTTA<br>TTTCTGTGCAGCAGACCGGGGTGAAAGT<br>TACTACTACACTCGACCCACCGAGTATG<br>AATTCTGGGGCCAGGGGACCCAGGTCAC<br>CGTCTCCTCA	QVQLVESGGGLVQAGGSLRLACIA<br>SGRTFHSYVMAWFRQAPGKEREFV<br>AAISWSSTPTYYGESVKGRFTISR<br>DNAKNTVYLQMNRLKPEDTAVYFC<br>AADRGESYYYTRPTEYEFWGQGTQ<br>VTVSS
H3	CAGGTGCAGCTGGTGGAGTCTGGGGGAG<br>GATTGGTGAAGACTGGGGGCTCTCTGAG<br>ACTCTCCTGTGCAGCCTCTGGCCGCACC<br>TTCAGTACCTACAGCATGGGCTGGTTCC<br>GCCAGGCTCCAGGGAAGGAGCGTGAGTT<br>TGTAGCAGGTATGCGCTGGACGGGTAGT<br>AGTACATTCTACTCAGACTCCGTGAAGG<br>GCCGATTCACCGTCTCCAGAAACAACGC<br>CAAGGACACGGTGTATCTGCACATGAAC<br>AGCCTGAAACCTGAGGACACGGCCGTTT<br>ATTACTGTGCAATCACGACTATCGTAAG<br>AGCTTACTATACTGAGTATACCGAAGCT<br>GACTTTGGTTCCTGGGGCCAGGGGACCC<br>AGGTCACCGTCTCCTCA	QVQLVESGGGLVKTGGSLRLSCAA<br>SGRTFSTYSMGWFRQAPGKEREFV<br>AGMRWTGSSTFYSDSVKGRFTVSR<br>NNAKDTVYLHMNSLKPEDTAVYYC<br>AITTIVRAYYTEYTEADFGSWGQG<br>TQVTVSS

Supplementary Table 2: DNA sequences of C1, C5, F2 and H3 nanobodies
