# Supplementary material for: A potent SARS-CoV-2 neutralising nanobody shows therapeutic efficacy in the Syrian golden hamster model of COVID-19
Source: Nat Commun. 2021 Sep 22;12:5469. doi: 10.1038/s41467-021-25480-z (PMC8458290; doi:10.1038/s41467-021-25480-z)
Supplement: Supplementary file 3 — Reporting summary [file 41467_2021_25480_MOESM3_ESM.pdf]

nature portfolio

Corresponding author(s): R J Owens, J H Nasimith

Last updated by author(s): Aug 5, 2021

Reporting Summary

Nature Portfolio wishes to improve the reproducibility of the work that we publish. This form provides structure for consistency and transparency in reporting. For further information on Nature Portfolio policies, see our Editorial Policies and the Editorial Policy Checklist.

Statistics

For all statistical analyses, confirm that the following items are present in the figure legend, table legend, main text, or Methods section.

n/a	Confirmed	
☐	☒	The exact sample size (n) for each experimental group/condition, given as a discrete number and unit of measurement
☐	☒	A statement on whether measurements were taken from distinct samples or whether the same sample was measured repeatedly
☐	☒	The statistical test(s) used AND whether they are one- or two-sided<br>*Only common tests should be described solely by name; describe more complex techniques in the Methods section.*
☒	☐	A description of all covariates tested
☒	☐	A description of any assumptions or corrections, such as tests of normality and adjustment for multiple comparisons
☐	☒	A full description of the statistical parameters including central tendency (e.g. means) or other basic estimates (e.g. regression coefficient) AND variation (e.g. standard deviation) or associated estimates of uncertainty (e.g. confidence intervals)
☐	☒	For null hypothesis testing, the test statistic (e.g. F, t, r) with confidence intervals, effect sizes, degrees of freedom and P value noted<br>*Give P values as exact values whenever suitable.*
☒	☐	For Bayesian analysis, information on the choice of priors and Markov chain Monte Carlo settings
☒	☐	For hierarchical and complex designs, identification of the appropriate level for tests and full reporting of outcomes
☒	☐	Estimates of effect sizes (e.g. Cohen's d, Pearson's r), indicating how they were calculated

Our web collection on statistics for biologists contains articles on many of the points above.

Software and code

Policy information about availability of computer code

Data collection	Xia2, RELION (v3.1), CRYOSPARC (v3.2)
Data analysis	SPR Sensorgrams were plotted using GraphPad Prism 8; ITC Data acquisition and analysis were performed using the Origin scientific graphing and analysis software package (v2021b, OriginLab) or AFFINImeter for global fitting of the displacement assay. RefMac (v5.0.32), Coot (v0.9.4.1), PHENIX (v1.19.2) and Relion (v3.1) software were used for the structure determinations. For in vivo studies Graph and statistical analysis were performed with Graphpad Prism 9 and Minitab version 16.For morphometric analysis, the HE-stained sections were scanned (NanoZoomer-XR C12000; Hamamatsu, Hamamatsu City, Japan) and analysed using the software programme Visiopharm (Visiopharm 2020.08.1.8403; Visiopharm, Hoersholm, Denmark).

For manuscripts utilizing custom algorithms or software that are central to the research but not yet described in published literature, software must be made available to editors and reviewers. We strongly encourage code deposition in a community repository (e.g. GitHub). See the Nature Portfolio guidelines for submitting code & software for further information.

Data

Policy information about availability of data

All manuscripts must include a data availability statement. This statement should provide the following information, where applicable:

- Accession codes, unique identifiers, or web links for publicly available datasets
- A description of any restrictions on data availability
- For clinical datasets or third party data, please ensure that the statement adheres to our policy

The coordinates and structure factors were deposited in the wwPDB with accession nos. C5 – RBD 7OAO [http://doi.org/10.2210/pdb, 7OAO/pdb] H3- RBD-C1 7OAP [http://doi.org/10.2210/pdb7OAP/pdb], F2–RBD 7OAY[http://doi.org/10.2210/pdb7OAY/pdb] C5-Alpha-RBD, 7OAU [http://doi.org/10.2210/pdb7OAU/pdb],

H3-Alpha RBD-C1 7OAQ[http://doi.org/10.2210/pdb7OAQ/pdb]. Spike C5 EM maps and models are deposited in the EMDB and wwPDB under accession codes, EMD-12777 [https://www.ebi.ac.uk/pdbe/entry/emdb/EMD-12777] and 7OAN [http://doi.org/10.2210/pdb7OAN/pdb]. Nanobody sequences are provided in the Supplementary Table 2. Source data are provided with this paper.Nanobody sequences are provided in the Supplementary Table 2.Source data are provided with this paper. The pOPINO vectors for producing nanobodies C1, C5, F2 and H3 have been deposited with Addgene (www.addgene.org) with IDs 171924, 171925, 171926, 171927. hCoV-19/Australia/VIC01/2020) GISAID accession number EPI_ISL_406844.

Field-specific reporting

Please select the one below that is the best fit for your research. If you are not sure, read the appropriate sections before making your selection.

[x] Life sciences [ ] Behavioural & social sciences [ ] Ecological, evolutionary & environmental sciences

For a reference copy of the document with all sections, see nature.com/documents/nr-reporting-summary-flat.pdf

Life sciences study design

All studies must disclose on these points even when the disclosure is negative.

Sample size	Sample size for animal studies (n =6/ group) was chosen to provide sufficient statistical power with minimal animal use.<br>The selection of groups of 6 animals was used based on a balanced one-way analysis of variance power calculation (significance level of 0.05 and power of 0.8):<br>k=2 (number of groups)<br>n=5.089995 (number of individuals per group)<br>f=1 (effect size - so will determine a 1-log reduction in viral titre)
Data exclusions	No data excluded
Replication	Biophysical assay measurements were successfully replicated in independent experiments (ITC x 3, SPR x2); virus neutralisation in vitro was carried out with four replicates; each animal study was carried out once with n =6 animals/group (see above)
Randomization	Animals were randomly assigned to each group for the in vivo studies. For in vitro biochemical assays randomization is not applicable as measurements are being made on a defined sample. The order of samples for the in vitro neutralisation assays was randomised
Blinding	The arms of the animal studies were blinded. The neutralisation assays were also blinded.

Reporting for specific materials, systems and methods

We require information from authors about some types of materials, experimental systems and methods used in many studies. Here, indicate whether each material, system or method listed is relevant to your study. If you are not sure if a list item applies to your research, read the appropriate section before selecting a response.

Materials & experimental systems

n/a	Involved in the study	
[ ]	[x]	Antibodies
[ ]	[x]	Eukaryotic cell lines
[x]	[ ]	Palaeontology and archaeology
[ ]	[x]	Animals and other organisms
[x]	[ ]	Human research participants
[x]	[ ]	Clinical data
[x]	[ ]	Dual use research of concern

Methods

n/a	Involved in the study	
[x]	[ ]	ChIP-seq
[x]	[ ]	Flow cytometry
[x]	[ ]	MRI-based neuroimaging

Antibodies

Antibodies used	rabbit anti-SARS-CoV nucleocapsid protein (1:6000, Rockland, 200-402-A50), rabbit anti-human Iba1/AIF1 (1:1000, Wako, 019-19741), rabbit anti-human prosurfactant protein-C (1:4000, SP-C; Abcam, ab40879), and mouse anti-human pan-cytokeratin (1:10000, clone PCK-26; Novus Biologicals, NB120-6401). EnVision+/HRP, Rabbit and Mouse respectively (undiluted ready-to-use reagent, Agilent K406311-2) HRP/Anti-M13 Monoclonal Conjugate (1:5000, Cytiva, 27-9421-01).red fluorescence labelled antibody (1:500, goat anti-rabbit 594; Invitrogen, A11012), second primary antibody (1:400, goat anti-human Iba1; Abcam, ab 5076), green fluorescence labelled antibody (1:500, donkey anti-goat 488; Invitrogen, A1105).
Validation	rabbit anti-SARS-CoV nucleocapsid protein (Rockland, 200-402-A50)<br>https://rockland-inc.com/store/Infectious-Disease-Antibodies-200-401-A50-O4L_3382.aspx<br>rabbit anti-human Iba1/AIF1 (Wako, 019-19741),<br>https://labchem-wako.fujifilm.com/us/product/detail/W01W0101-1974.html

rabbit anti-human prosurfactant protein-C (Abcam, ab40879<br>https://www.abcam.com/prosurfactant-protein-c-antibody-ab40879.html<br>mouse anti-human pan-cytokeratin (Novus Biologicals, NB120-6401<br>https://www.novusbio.com/products/cytokeratin-pan-antibody-pck-26_nb120-6401<br>HRP/Anti-M13 Monoclonal Conjugate, Cytiva, 27-9421-01<br>https://uk.vwr.com/assetsvc/asset/en_GB/id/9458946/contents<br>goat anti-human Iba1; Abcam, ab 5076<br>https://www.abcam.com/iba1-antibody-ab5076.html

Eukaryotic cell lines

Policy information about cell lines

Cell line source(s)	expi 293 (Thermofisher) and Vero/hSLAM cells [ECACC 04091501]. Vero 76 clone e6 cells [ECACC 85020206]
Authentication	cells were bought from a commercial supplier or ECACC, no independent authentication check was made
Mycoplasma contamination	cells certified mycoplasma negative by supplier
Commonly misidentified lines<br>(See ICLAC register)	no commonly miss-identfiied cell lines used in the study

Animals and other organisms

Policy information about studies involving animals; ARRIVE guidelines recommended for reporting animal research

Laboratory animals	Golden Syrian hamsters (male 8-10 weeks old from Janvier Labs, France) and (males and females 7-9 weeks old from Envigo, London, UK).
Wild animals	no wild animals were used in the study
Field-collected samples	no field collected samples were used in the study.
Ethics oversight	All experimental work was conducted under the authority of a UK Home Office approved project license that had been subject to Local ethical review at PHE Porton Down by the Animal Welfare and Ethical Review Body (AWERB) as required by the 'Home Office Animals (Scientific Procedures) Act 1986'. Animal work was approved by the local University of Liverpool Animal Welfare and Ethical Review Body and performed under UK Home Office Project Licence PP4715265.

Note that full information on the approval of the study protocol must also be provided in the manuscript.
